# Black Plane Solutions and Localized Gravitational Energy

**DOI:** 10.1155/2015/109329

**Published:** 2015-08-06

**Authors:** Paul Halpern, Jennifer Roberts

**Affiliations:** Department of Mathematics, Physics and Statistics, University of the Sciences in Philadelphia, 600 S. 43rd Street, Philadelphia, PA 19104, USA

## Abstract

We explore the issue of gravitational energy localization for static plane-symmetric solutions of the Einstein-Maxwell equations in 3+1 dimensions with asymptotic anti-de Sitter behavior. We apply three different energy-momentum complexes, the Einstein, Landau-Lifshitz, and Møller prescriptions, to the metric representing this category of solutions and determine the energy distribution for each. We find that the three prescriptions offer identical energy distributions, suggesting their utility for this type of model.

## 1. Introduction

Ever since Einstein developed his field equations of general relativity, defining gravitational energy and momentum in a consistent manner has been a perplexing conundrum. While other forms of energy and momentum can be defined locally through the stress-energy tensor, gravitational energy does not readily lend itself to a localized, tensorial description. Einstein's proposed resolution of the idea is an energy-momentum pseudotensor constructed from the metric components [1].

While the Einstein energy-momentum complex offers the important feature that conservation laws are well defined for both energy and momentum, it has the disadvantage of being asymmetric in its indices, making it difficult to define a conserved angular momentum. Moreover, the complex is highly dependent on the coordinate system used, favoring the use of quasi-Cartesian (small perturbations of a flat background) coordinates.

To mitigate these difficulties, several other theorists have offered alternative ways to construct energy-momentum complexes. These include prescriptions by Landau and Lifshitz [2], Papapetrou [3], Tolman [4], Møller [5, 6], Weinberg [7], and others. These complexes each conserve angular momentum in addition to energy momentum. Møller's prescription offers the additional advantage of being independent of the coordinate system used.

Despite the diversity of energy-momentum complexes, there are a wide range of solutions to the Einstein equations for which they exhibit identical results. Virbhadra first suggested this possibility in 1990 when he demonstrated how different complexes applied to the same metric yielded identical distributions [8]. In 1996, Aguirregabiria et al. demonstrated that all metrics of the Kerr-Schild class, including the Schwarzchild, Reissner-Nordström, and other well-known solutions, produced the same energy and momentum distributions for a variety of prescriptions [9]. Three years later, Virbhadra generalized this result to metrics outside the Kerr-Schild class, contingent on the use of Kerr-Schild Cartesian coordinates [10].

In recent years, many researchers have applied various energy-momentum complexes to a wide range of generalizations of black hole solutions and found consistent results. Vagenas has investigated the energy distribution in the dyadosphere of a Reissner-Nordström black hole [11]. Aydogdu and Salti have found the energy content of Bianchi-type I cosmologies in Møller's tetrad model of gravity [12]. Berman has examined the energy and angular momentum of dilation black holes [13]. Radinschi et al., along with various collaborators, have determined the energy-momentum distribution for Ho$\hat{r}$ava-Lifshitz black holes [14], stringy black holes [15, 16], charged black holes in generalized dilaton-axion gravity [17], asymptotic de Sitter spacetimes [18], and a Schwarzschild-quintessence spacetime [19]. Sharif and Amir have investigated the energy-momentum distribution of spatially homogeneous, rotating spacetimes in teleparallel gravity [20]. Sharif and Jawad have calculated the energy of rotating spacetimes in teleparallel gravity [21]. Xulu has examined the Møller energy of nonstatic, spherically symmetric spacetimes [22]. Halpern has investigated the energy distribution of a charged black hole with a minimally coupled scalar field [23] and, along with Pecorino, a static, electrically neutral, spherically symmetric massive body embedded in a space filled with phantom energy [24].

In this paper, we consider black plane solutions. Developed by Cai and Zhang [25], these represent static, plane-symmetric solutions of the Einstein-Maxwell equations with a negative cosmological constant. Cai and Zhang determined that these possess a causal structure similar to the Reissner-Nordström solution, but with a Hawking temperature that varies with *M* ^1/3^, where *M* is the ADM mass density. They located inner and outer event horizons for these solutions, identifying how their locations depend on the objects' mass density, charge density, and cosmological constant. Wu et al. explored the global structure of such flat topologies [26].

It is interesting to determine the localized energy distribution of black plane solutions. One of us investigated these using the Einstein prescription [27]. We extend these results by also applying the Landau-Lifshitz and Møller prescriptions and comparing the three results.

## 2. Applying Einstein's Prescription to Black Plane Solutions

We now apply Einstein's prescription to the black plane metric, with asymptotic anti-de Sitter behavior. As mentioned, this prescription is coordinate-dependent, favoring the use of quasi-Cartesian (small perturbations of a flat background) coordinates. Although this model is asymptotically anti-de Sitter, by assuming a small-enough cosmological constant, the manifold is sufficiently close to flat in the vicinity of the black plane for this prescription to be valid. Following Cai and Zhang's notation, we can express this as

(1) $$\begin{array}{c} ds^{2}=A\left(\;r\;\right)dt^{2}-A^{-1}\left(\;r\;\right)dr^{2}+\frac{\Lambda }{3}r^{2}\left(\;dx^{2}+dy^{2}\;\right), \end{array}$$

where

(2) $$\begin{array}{c} A\left(\;r\;\right)=-\frac{\Lambda }{3}r^{2}-\frac{m}{r}+\frac{q^{2}}{r^{2}} \end{array}$$

with

(3) $$\begin{array}{c} m=-\frac{12\pi M}{\Lambda },\;q=2\pi Q. \end{array}$$

Here, *M* is the ADM mass, *Q* is the electric charge, and Λ is a negative cosmological constant. Because of the reflection symmetry with respect to the plane *z* = 0, we have taken *r* = |*z*|.

Now let us use Einstein's energy-momentum complex to calculate the energy distribution of this solution. In Einstein's prescription, the energy-momentum complex is given by

(4) $$\begin{array}{c} {\theta_{i}}^{k}=\frac{1}{16\pi }{{H_{i}}^{kl}}_{,l}, \end{array}$$

where the superpotentials *H*_*i*_^*kl*^ are

(5) $$\begin{array}{c} {H_{i}}^{kl}=\frac{g_{in}}{\sqrt{-g}}{\left[\;-g\left(\;g^{kn}g^{lm}-g^{ln}g^{km}\;\right)\;\right]}_{,m} \end{array}$$

with the antisymmetric property that

(6) $$\begin{array}{c} {H_{i}}^{kl}=-{H_{i}}^{lk}. \end{array}$$

The relevant components of *H*_*i*_^*kl*^ are

(7) $$\begin{array}{c} {H_{0}}^{03}={H_{0}}^{30}=\frac{\Lambda \left(q^{2}-mr-r^{4}\left(\Lambda /3\right)\right)}{12\pi r} \end{array}$$

with the other components of *H*_*i*_^*kl*^ = 0.

The energy-momentum components are found by the volume integral:

(8) $$\begin{array}{c} P_{i}=\int \int \int {\theta_{i}}^{0}dx^{1}dx^{2}dx^{3}. \end{array}$$

Using Gauss' theorem, we can rewrite this as a surface integral:

(9) $$\begin{array}{c} P_{i}=\frac{1}{16\pi }\int \int {H_{i}}^{0\alpha }\mu_{\alpha }dS, \end{array}$$

where *μ*_*α*_ is the outward unit vector normal to the surface element *dS*.

We select the surface element to be a planar shell with fixed *r* (and hence two fixed values of *z*); then *dS* = *dxdy* and *μ*_*α*_ is the unit radial vector.

Therefore the energy density is

(10) $$\begin{array}{c} E=P_{0}=\frac{{H_{0}}^{30}}{16\pi }. \end{array}$$

Finally, we substitute expression (7) into this relationship and find the energy distribution according to Einstein's prescription applied to the black plane solution to be

(11) $$\begin{array}{c} E=M+\frac{\pi \Lambda Q^{2}}{3r}-\frac{\Lambda^{2}r^{3}}{36\pi }. \end{array}$$

## 3. Evaluating the Energy Using the Landau-Lifshitz Prescription

Let us now compare our results to those of a second technique for determining the local energy, the Landau-Lifshitz prescription, which similarly relies on quasi-Cartesian coordinates. However, once again, by assuming a sufficiently small cosmological constant, the manifold is close enough to flat in the vicinity of the black plane for this prescription to be valid. It can be expressed as

(12) $$\begin{array}{c} L^{ab}=\frac{1}{16\pi }l_{,cd}^{acbd}, \end{array}$$

where

(13) $$\begin{array}{c} l^{acbd}=-g\left(\;g^{ab}g^{cd}-g^{ad}g^{cb}\;\right). \end{array}$$

Inserting the metric components for Cai and Zhang's solution (1), we obtain the only relevant nonzero components to be

(14) $$\begin{array}{c} l^{0003}=l^{0300}=\frac{\Lambda \left(q^{2}-mr-r^{4}\left(\Lambda /3\right)\right)}{12\pi r}. \end{array}$$

Again, we employ Gauss' theorem to express the total energy as a surface integral:

(15) $$\begin{array}{c} E=\frac{1}{16\pi }\int \int l^{000\alpha }\mu_{\alpha }dS. \end{array}$$

Finally, we find the energy distribution according to Landau and Liftshitz's prescription applied to the black plane solution to be

(16) $$\begin{array}{c} E=M+\frac{\pi \Lambda Q^{2}}{3r}-\frac{\Lambda^{2}r^{3}}{36\pi }. \end{array}$$

This is identical to the result obtained using the Einstein energy-momentum complex.

## 4. Applying the Coordinate-Independent Prescription of Møller

We now turn to a third method for determining the local energy, the Møller prescription, which has the marked advantage of being coordinate-system-independent. The complex defined by Møller can be expressed as

(17) $$\begin{array}{c} \Xi_{i}^{k}=\frac{1}{8\pi }\chi_{i,p}^{kp}, \end{array}$$

where

(18) $$\begin{array}{c} \chi_{i}^{kl}=\sqrt{-g}\left[\;g_{ip,q}-g_{iq,p}\;\right]g^{kq}g^{lp}. \end{array}$$

Inserting the metric components for Cai and Zhang's solution (1), we obtain the only nonzero component to be

(19) $$\begin{array}{c} {\chi_{0}}^{03}={\chi_{0}}^{30}=\frac{\Lambda \left(q^{2}-mr-r^{4}\left(\Lambda /3\right)\right)}{12\pi r}. \end{array}$$

One more time, we employ Gauss' theorem to express the total energy as a surface integral:

(20) $$\begin{array}{c} E=\frac{1}{16\pi }\int \int {\chi_{0}}^{0\alpha }\mu_{\alpha }\;dS. \end{array}$$

Integrating over the full range of coordinates, we find the total energy using the Møller complex to be

(21) $$\begin{array}{c} E=M+\frac{\pi \Lambda Q^{2}}{3r}-\frac{\Lambda^{2}r^{3}}{36\pi }. \end{array}$$

This is identical to the expression obtained using the prescriptions of Einstein and Landau-Lifshitz.

## 5. Conclusion

We have determined the localized energy distribution for black plane solutions by means of Einstein's, Landau and Lifshitz's, and Møller's prescriptions for the energy-momentum complexes and shown that all three yield identical results. We found this quantity to be well defined and well behaved and, importantly, consistent throughout. Our findings help support the utility of energy-momentum complexes as consistent, well-defined ways of describing the local distribution of gravitational energy.
